# Peptide-Mediated Liposomal Doxorubicin Enhances Drug Delivery Efficiency and Therapeutic Efficacy in Animal Models

**DOI:** 10.1371/journal.pone.0083239

**Published:** 2013-12-27

**Authors:** De-Kuan Chang, Pi-Chun Li, Ruei-Min Lu, Wann-Neng Jane, Han-Chung Wu

**Affiliations:** 1 Institute of Cellular and Organismic Biology, Academia Sinica, Taipei, Taiwan; 2 Institute of Plant and Microbial Biology, Academia Sinica, Taipei, Taiwan; H. Lee Moffitt Cancer Center & Research Institute, United States of America

## Abstract

Lung cancer ranks among the most common malignancies, and is the leading cause of cancer-related mortality worldwide. Chemotherapy for lung cancer can be made more specific to tumor cells, and less toxic to normal tissues, through the use of ligand-mediated drug delivery systems. In this study, we investigated the targeting mechanism of the ligand-mediated drug delivery system using a peptide, SP5-2, which specifically binds to non-small cell lung cancer (NSCLC) cells. Conjugation of SP5-2 to liposomes enhanced the amount of drug delivered directly into NSCLC cells, through receptor-mediated endocytosis. Functional SP5-2 improved the therapeutic index of Lipo-Dox by enhancing therapeutic efficacy, reducing side effects, and increasing the survival rate of tumor-bearing mice in syngenic, metastatic and orthotopic animal models. Accumulation of SP5-2-conjugated liposomal doxorubicin (SP5-2-LD) in tumor tissues was 11.2-fold higher than that of free doxorubicin, and the area under the concentration-time curve (AUC_0–72 hours_) was increased 159.2-fold. Furthermore, the experiment of bioavailability was assessed to confirm that SP5-2 elevates the uptake of the liposomal drugs by the tumor cells *in vivo*. In conclusion, the use of SP5-2-conjugated liposomes enhances pharmacokinetic properties, improves efficacy and safety profiles, and allows for controlled biodistribution and drug release.

## Introduction

Lung cancer continues to be the leading cause of cancer deaths in the United States, and a major cause of death worldwide [1], [2]. The two main types of lung cancer are non-small cell lung cancer (NSCLC) and small cell lung cancer (SCLC). Approximately 80% of all primary pulmonary tumors are of the NSCLC type [3]; these have a limited response rate to current chemotherapeutic agents, and afflicted patients have a two-year survival rate of 21% [4]. A major factor contributing to poor chemotherapeutic efficacy in the treatment of lung cancer is the high interstitial fluid pressure (IFP) within tumors. Tumor blood vessels are irregular and tortuous, with abnormal structures that have leaky and heterogeneous vessel walls [5], [6], [7]. Immature vasculature and high IFP hinder drug delivery into solid tumors, and reduce the therapeutic efficacy of systemically administered chemotherapy [8], [9]. Ultimately, only 5–10% of drugs accumulated in normal viscera enter into tumor tissues [10], [11].

A possible strategy to overcome these limitations is to link anti-cancer drugs to monoclonal antibodies (mAbs) or peptides that bind to uniquely- or over-expressed antigens or receptors on cancer cells. To date, various ligand-targeted therapeutics, such as immunotoxins, antibody drug-conjugates, and ligand-targeted liposomes, have been developed [12], [13], [14]. A rapidly developing class of ligand-targeted therapeutics is the ligand-targeted liposome, which combines the advantages of passive targeting (liposome) with active tumor-specific targeting (ligand) functions. Liposomal-based phospholipid bilayer vesicles are effective particulate drug carriers, due to their abilities to improve the pharmacokinetic and pharmacodynamic profiles of the underlying drugs. In addition, remote loading methods (such as the ammonium sulfate method) can encapsulate 15,000 doxorubicin molecules into a single liposome with greater than 95% efficiency [15], [16]. Several liposomal formulations have received clinical approval [17], including liposomal doxorubicin (CAELYX™/Doxil®) [18], which has been widely studied for the treatment of various cancers [17], [19], [20]. Conjugation of ligands to liposomes enables active targeting of the tumor through binding to tumor-associated antigens or receptors, resulting in enhanced and more selective anticancer activity [21], [22], [23], [24], [25], [26].

Peptides are considered promising candidates for guiding liposomes to target against tumors, which helps to improve the therapeutic index of liposome-containing drugs by increasing their selectivity and specificity [22], [27], [28]. Indeed, peptide-mediated tumor-targeting is a key area of research into cancer-targeted therapy at present. Chang et al. isolated several novel peptides, including SP5-2, capable of binding to non-small cell lung cancer (NSCLC) cell lines [11]. Targeting peptides were able to recognize NSCLC surgical specimens, and coupling of peptide to liposomes carrying the drugs, such as vinorelbine or doxorubicin, markedly increased the therapeutic index and survival rate in xenograft animal models [11]. Elucidation of the exact targeting mechanisms is critical for development of this drug delivery system. In this study, we systematically investigated the mechanisms underlying the delivery of SP5-2-mediated liposomal doxorubicin (SP5-2-LD) to tumor cells. We report that SP5-2-LD increases pharmacokinetic of drugs and enhances drugs accumulation in both tumor tissues and inside of the cancer cells through endocytosis, thereby enhancing the therapeutic efficacy in syngenic, metastatic, and orthotopic animal models.

## Materials and Methods

### Cell lines and cultures

The human non-small cell lung carcinoma cell line NCI-H460 (ATCC: HTB-177), and murine Lewis Lung Carcinoma cell line LL/2 (ATCC: CRL-1642), were used in this study. H460 cells were grown in RPMI 1640 (Gibco, Carlsbad, CA) containing 10% fetal bovine serum (FBS, Gibco) at 37°C in a 5% CO_2_ incubator. LL/2 cells were grown in DMEM (Gibco) containing 10% FBS at 37°C in a 10% CO_2_ incubator.

### Preparation of synthetic peptide-conjugated liposomes containing doxorubicin or sulforhodamine B

The synthetic targeting peptide SP5-2 (TDSILRSYDWTY) and mutant peptide MP5-2 (TDSILRSYDGGG) were synthesized and purified by reverse phase high performance liquid chromatography to >95% purity at Academia Sinica (Taipei, Taiwan). Peptide-conjugated liposomes containing doxorubicin or sulforhodamine B (SRB, Sigma, St. Louis, MO) were prepared as described previously [27]. Briefly, the peptide was coupled to NHS-PEG-DSPE [N-hydroxysuccinimido-carboxyl-polyethylene glycol (MW, 3400)-derived distearoylphosphatidyl ethanolamine] (NOF Corporation, Tokyo, Japan) at a 1∶1.5 molar ratio. The reaction was completed and confirmed by quantitation of the remaining amino groups using TNBS (Trinitrobenzenesulfonate) reagent (Sigma). Doxorubicin was encapsulated in liposomes through a remote loading method at a concentration of 1 mg of drug per 10 µmol phospholipids. Phosphatidylethanolamine-dioleoyl-sulforhodamine B was loaded into liposomes by incorporation with the vesicular membrane, at a concentration of 1 mg of drug per 44 µmol phospholipids. Peptidyl-PEG-DSPE was transferred to pre-formed liposomes by co-incubation at the transition temperature of the lipid bilayer. Each targeting liposome had a particle size ranging from 65 to 75 nm in diameter and contained 500 peptide molecules, as described previously [11], [22], [28], [29].

### Endocytosis of liposome conjugates with SP5-2 by H460 cells

H460 lung cells were incubated at 37°C or 4°C with SP5-2-conjugated liposomal SRB (SP5-2-LS), MP5-2-LS, or LS. After 30 min of incubation, the cells were washed with PBS, stained with DAPI (Vector Laboratories, Burlingame, CA), and examined with a TCS-SP5-AOBS Leica confocal microscope (Leica Microsystems). The images were merged using the Leica Application Suite (LAS) software (Leica, Bannockburn, IL). For internalization analysis, H460 cells were incubated at 37°C with 2.5 or 10 µg/ml of SP5-2-conjugated liposomal doxorubicin (SP5-2-LD) or LD. After 15- or 60-min of incubation, the cells were washed with PBS, acid glycin buffer and were lysed with 1% Triton X-100. After centrifugation, the doxorubicin concentration was detected using spectrofluorometry at λ_Ex/Em_ 485/590 nm (Synergy HT Multi-Detection Microplate Reader, BioTek Instruments, Winooski, VT) and was calculated by interpolation using a standard curve.

### Analysis of SP5-2-LD uptake by transmission electron microscopy

H460 cells were plated in 100-mm tissue culture dishes (Becton Dickinson, Bedford, MA) (500,000 cells per dish) in 10 ml of growth media, for 24 h prior to the experiment. Cells were incubated with SP5-2-LD or MP5-2-LD (2 mg/ml) at 37°C for 5 min, and then were washed twice with PBS. Cells were harvested by trypsinization before being fixed using a high-pressure freezer (Lecia EM PACT2). Samples were transferred to an acetone (containing 2.5% glutaraldehyde) solution for 3–5 days at −85°C, and were then shifted to a series of temperatures (−60, −20, 0, and 4°C) for a set period of time (24, 24, 6 and 1 hour, respectively). Fixed samples were then soaked overnight in a 3∶1, 1∶1, and 1∶3 ratio of acetone and Spurr embedding resin (Polysciences, Inc., Warrington, PA), and finally in pure Spurr resin. The resin-embedded cells were prepared using the Leica Automatic Freeze-Substitution System and visualized using a transmission electron microscope.

### Quantum dots (Qdots) labeled by synthetic peptides

Qdot 800 ITK amino PEG quantum dots (Invitrogen, Carlsbad, CA) were used to investigate *in vitro* cell binding. The procedures for synthesis of ligand-conjugated Qdots were modified from a previous report [30]. Briefly, Qdots were conjugated with sulfo-SMCC (Sulfosuccinimidyl-4-(N-maleimidomethyl)cyclohexane-1-carboxylate; Pierce) to generate a maleimide-activated surface on the Qdots, and free sulfo-SMCC was removed by a NAP-10 desalting column (GE healthcare, San Francisco, CA). Peptides were reduced by TCEP to generate an activated thiol group in the carboxyl terminus, and were subsequently reacted with the maleimide-functionalized Qdots at 4°C overnight. Peptide-conjugated Qdots were purified using sepharose-4B gel filtration chromatography and eluted with HEPES buffer. The concentration of Qdots was examined by spectrofluorometry and calculated by interpolation using a standard curve. Peptide-coated Qdots were stained in LL/2 cells for 1 hour, washed with PBS three times, stained with DAPI, and then visualized by fluorescent microscopy.

### Flow cytometry

The analysis of cell surface molecules on lung cancer cell line was determined using a fluorescein isothiocyanate (FITC)-conjugated peptides. Cells were incubated with 1, 3, 9, and 27 µg/ml FITC-peptides at 4°C for 1 hour. After three times wash with cold PBS, cells were detected and analyzed with flow cytometry.

### Peptide-binding and competition assay

H460 cells were seeded in 96-well ELISA plates at 1×10^4^ per well. After 24-hour incubation, cells were fixed with 3.7% paraformaldehyde for 10 min at 4°C and then blocked with 1% BSA in PBS for 1 hour at room temperature. Serial diluted biotinylated peptides were incubated with cells for 1 hour at room temperature. After twice washing with PBS, cells were incubated with HRP-conjugated streptavidin (1∶5000 dilution) for 1 hour and then washed with PBS supplemented with 0.1% tween-20 for four times. Binding activity was assessed by cellular ELISA with optical density at 490 nm. For competition assay, cells were firstly incubated with unlabeled peptides at room temperature for 1 hour before incubated with 10 µg/ml biotinylated SP5-2 peptides.

### In vivo homing experiments and tissue distribution of phages

C57BL/6 mice (6-week-old, National Laboratory Animal Center (NLAC), Taipei, Taiwan) were injected s.c. in the dorsolateral flank with 2×10^6^ LL/2 cells in 100 µL serum-free media. The mice bearing size-matched lung cancer xenografts (approximately 500 mm^3^) were injected i.v. with 10^9^ pfu of the targeting PC5-2, or control helper phage. After perfusion, xenograft tumors and mouse organs were removed and homogenized. The phages bound to each tissue sample were recovered through the addition of ER2738 bacteria and titered on IPTG/X-Gal agar plates. All the animal studies were carried out following strict guidelines form the care and use manual of National Laboratory Animal Center. The protocol was approved by the Committee on the Ethics of Animal Experiments of Academia Sinica (Permit Number: MMi-ZOOWH2009102). The mice were killed with 50% CO_2_ containing 50% O_2_.

### Syngenic animal model

C57BL/6 mice (6-week-old, NLAC) were injected s.c. in the dorsolateral flank with 2×10^6^ LL/2 cells in 100 µL serum-free media. Mice with size-matched tumors (approximately 50–100 mm^3^) were then randomly assigned to different treatment groups and injected with SP5-2-LD, MP5-2-LD, LD, FD or saline through the tail vein. The dosage of SP-5-2-LD was 1 or 2 mg/kg, injected twice a week for four weeks. Mouse body weight and tumor size were measured twice a week. Tumor volumes were calculated using the equation: length × (width)^2^ ×0.52 and presented as standard error of the mean. This study used humane endpoint by determining the tumor size (>10% of body weight) or mouse weight loss (>20% of body weight). Mice were observed twice a week and given soft diet. Animal care was carried out in accordance with the guidelines of Academia Sinica, Taiwan.

### Lung metastasis animal model

LL/2 cells were washed twice by centrifugation in media without serum at room temperature, and re-suspended in media at 4°C prior to injection. Cell viability was determined by trypan blue exclusion, and only cell suspensions with greater than 95% viability and without cell clumping were used. Cells (1×10^5^ cells/mouse) were inoculated via the lateral tail vein of 6-week-old female C57BL/6 mice. Mice were then treated with different formulations of anti-cancer drugs. The dosage was 1 or 2 mg/kg twice a week for four weeks. Mouse body weight and survival rate were measured twice a week. The endpoint was determined by the mouse weight loss (>20% of body weight) or mouse activity assessment (hunching, stationary, ruffling and poor grooming). Mice were observed every day and given soft diet. Animal care was carried out in accordance with the guidelines of Academia Sinica, Taiwan.

### Orthotopic lung adenocarcinoma model

SCID mice (4-week-old) were anesthetized with isoflourane mixed with oxygen and placed in the right lateral decubitus position. The skin overlying the left chest wall in the mid-axillary line was treated with alcohol, and the underlying chest wall and intercostal spaces were visualized. H460 cells (5×10^5^; single-cell suspensions of over 90% viability as determined by trypan blue exclusion) in 50 µL serum-free media plus Matrigel Matrix (BD Biosciences) (1∶1) were injected into the left lateral thorax, at the lateral dorsal axillary line. After tumor injection, the mice were turned to the left lateral decubitus position and observed for 45 to 60 minutes until fully recovered. Mice were then treated with different formulations of anti-cancer drugs. The dosage was 1 mg/kg twice a week for four weeks. Mouse body weight and survival rate were measured twice a week. The endpoint was determined by the mouse weight loss (>20% of body weight) or mouse activity assessment (hunching, stationary, ruffling and poor grooming). Mice were observed every day and given soft diet. Animal care was carried out in accordance with the guidelines of Academia Sinica, Taiwan.

### Pharmacokinetic and biodistribution studies

Human cancer xenografts were established in NOD/SCID mice (NLAC). H460 cells (5×10^6^) were injected subcutaneously (s.c.) into the dorsolateral flanks of 4-week-old mice. Mice bearing H460 xenografts (∼500 mm^3^) were injected intravenously (i.v.) with various formulations of liposomal doxorubicin (SP5-2-LD, MP5-2-LD, and LD) and free doxorubicin at a dose of 2 mg/kg. At selected time points, three mice in each group were anaesthetized and sacrificed with 50% CO_2_ containing 50% O_2_. Blood samples were collected through submaxillary punctures, and plasma samples were prepared. After perfusion, xenograft tumors and mouse organs were removed and homogenized. Tumor cell nuclei were isolated and nuclear doxorubicin was extracted as described previously [31], [32]. The total doxorubicin concentration was measured as described by Mayer et al. [32]. Total doxorubicin was quantified using spectrofluorometry at λ_ex_ 485/20 nm and λ_em_ 645/40 nm (Synergy HT Multi-Detection Microplate Reader, BioTek Instruments, Winooski, VT).

To correct for background fluorescence, a standard curve was obtained by spiking tissue extracts derived from mice that did not receive doxorubicin. Tissue concentrations of doxorubicin were expressed as microequivalents per milliliter of plasma or per gram of tissue. A standard doxorubicin curve was prepared in control homogenates by following the above extraction procedure. Drug levels were estimated on the basis of doxorubicin fluorescent equivalents.

### Statistical analyses

We analyzed phage titer, tumor volume and body weight data using two-sided unpaired Student's *t*-test and doxorubicin concentration data using ANOVA. We considered a *P* value below 0.05 as significant for all analyses. Values are represented as mean ± standard deviation (S.D.).

## Results

### Effects of SP5-2 on binding and uptake of liposomes by cultured cells

**Figure 1:**
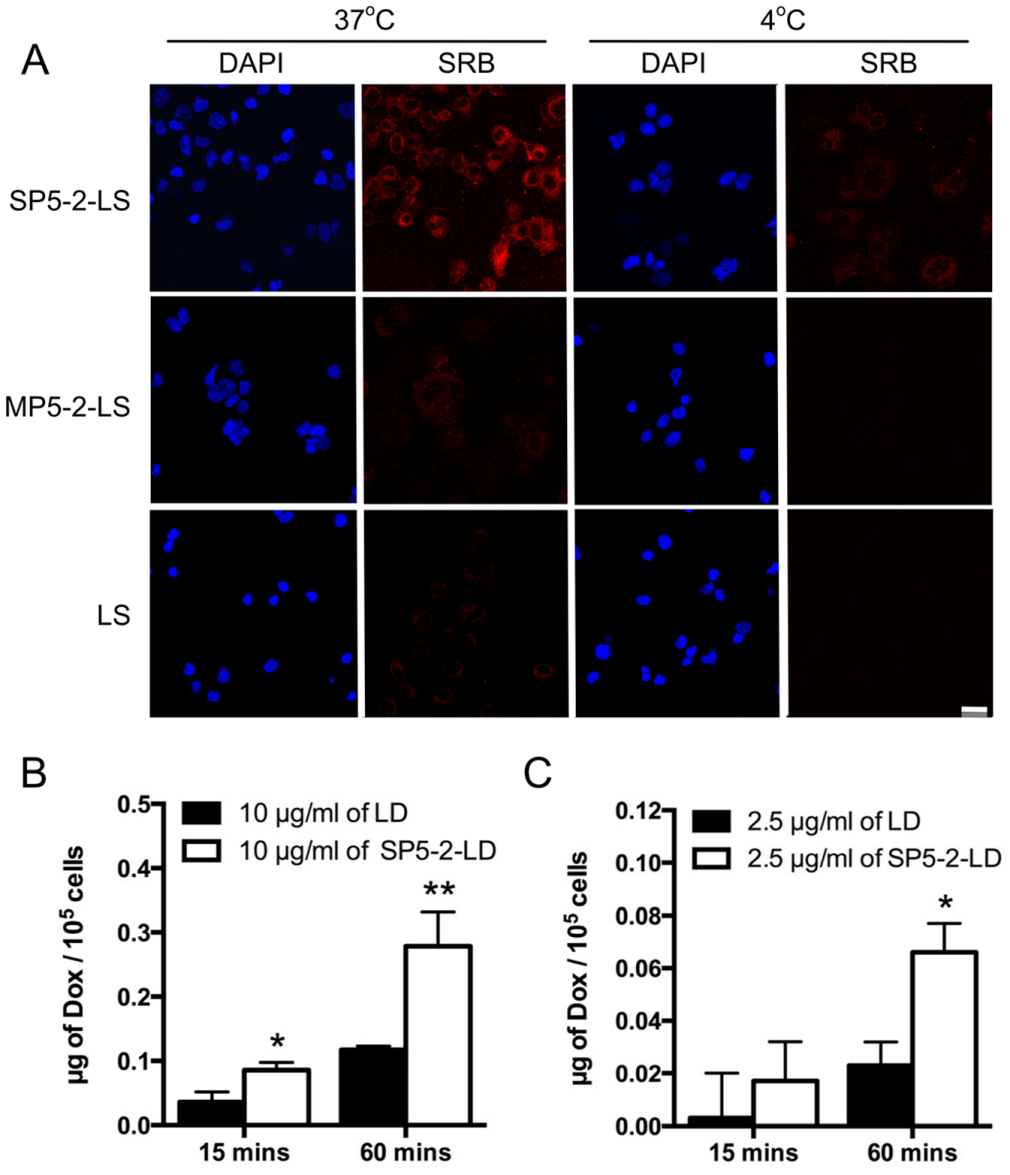
SP5-2 enhances internalization of liposomes. (A) Fluorescence microscopy was used to visualize H460 cells after incubation with either SP5-2-liposomal SRB (SP5-2-LS), MP5-2-LS or LS at the indicated temperatures. Nuclear staining was performed using DAPI (Scale bar: 30 µm). Internalization studies of SP5-2-LD and LD in H460 cells were incubated with 10 µg/ml (B) or 2.5 µg/ml (C) drugs at 37°C, respectively. Doxorubicin uptake by the cells was quantified at the indicated times, following the removal of surface-bound liposomal drugs.

Sulforhodamine B (SRB) is a non-titratable membrane impermeable fluorophore [33] and can bind electrostatically on basic amino acid residues of protein under mild acidic conditions [34]. Its sensitivity is comparable with that of several fluorescence dyes and is nondestructive and indefinitely stable in cells [34]. These practical advances make SRB an appropriate and sensitive material to investigate the mechanism of substrate release from encapsulated liposomes. To track the fate of liposome contents following its binding to cells, SRB-encapsulated liposomes were used to perform the binding and uptake assay [35]. These liposomes were further conjugated with SP5-2 to form targeting SP5-2-labeled liposomal SRB (SP5-2-LS). Upon co-incubation, SP5-2-LS binds to the plasma membrane of H460 cells, resulting in the uptake of the SP5-2-LS by the cell. The SRB will be released by broken down the liposomes and activated within the low pH environment of the endosomal and lysosomal compartments [16], [34]. To simultaneously and directly measure both the endocytosed liposomes and the intracellular locations of SRB, a confocal microscopy will be used in this study. Our result showed that incubation of H460 cells with SP5-2-LS at 37°C for 30 minutes resulted in cytoplasmic fluorescence surrounding the nuclei (Figure 1). However, such internalization of Lipo-SRB fluorescence via SP5-2 was not observed when H460 cells were incubated with SP5-2-LS at 4°C, or with either non-targeting Lipo-SRB (LS) or mutant SP5-2-Lipo-SRB (MP5-2-LS) at 37°C (Figure 1A). Furthermore, no specific fluorescence could be detected in H460 cells when cells were incubated with either LS or MP5-2-LS at 4°C (Figure 1A).

In addition, we conjugated SP5-2 to Liposomal doxorubicin (SP5-2-LD) and measured the dosage of drug uptake by tumor cells. As shown in Figure 1B, the drug dosage for SP5-2-LD-treated tumor cells was significantly higher than that for LD-treated tumor cells. Interestingly, when we decreased the drug concentration, it also showed that SP5-2 helped to enhance the accumulation of doxorubicin in tumor cells (Figure 1C). These data suggest that SP5-2 could facilitate the increase in drug uptake by the lung cancer cells.

### Enhanced tumor drug delivery of SP5-2-conjugated liposomes through cell endocytosis

**Figure 2:**
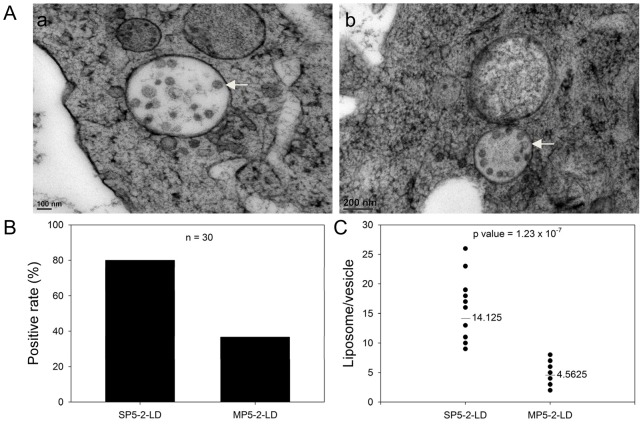
Transmission electron microscopy reveals SP5-2-mediated internalization of liposomal doxorubicin in H460 cells. (A) Transmission electron micrographs of H460 cells at 37°C, following a 10-minute incubation with SP5-2-LD (final doxorubicin concentration of 2 µg/ml). Arrows indicate endosome-containing liposomes. (B) Percentage of cells containing internalized liposomes (positive rate), after incubation with either SP5-2-LD or MP5-2-LD. (C) The number of liposomes per vesicle were calculated using randomly selected endosomes in H460 cells (n = 30 in each group, P  = 1.23×10^−7^).

The entry routes of liposomes in target cells refer to receptor-mediated endocytosis, lipid-mediated poration and lipid fusion [36], [37], [38], which accomplish the passive targeting feature of liposomes. Although our targeting peptide, SP5-2, enhanced the uptake of liposomes in NSCLC cells, the delivery mechanism is still not determined. In order to verify the accumulation of intracellular drugs by SP5-2-conjugated liposomes through endocytosis, we used transmission electron microscopy. Studies of membrane traffic between the cytoplasm and surface of a cell show that endocytosis can occur within a minute or less [39], [40], [41]. To understand the SP5-2-conjugated liposomes enhanced drug delivery through receptor-mediated endocytosis, tumor cells were incubated with SP5-2-conjugated liposomal doxorubicin (SP5-2-LD) at 37°C for 5 minutes. As shown in Figure 2, our data revealed that most liposomes were endocytosed by tumor cells and then were encapsulated in the endosomes (Figure 2A, arrows). Moreover, we calculated the number of cells with encapsulated liposomes (positive cells) and found that the positive rate of cellular endocytosis was 80% in the SP5-2-LD group (Figure 2B), but only 36.7% in the MP5-2-LD group. We also determined the mean number of liposomes in each vehicle, and found it to be 3-fold higher in the SP5-2-LD group as compared to the MP5-2-LD group (Figure 2C). These results indicate that SP5-2 directly enhances the delivery of liposome particles into cancer cells through receptor-mediated endocytosis.

**Figure 3:**
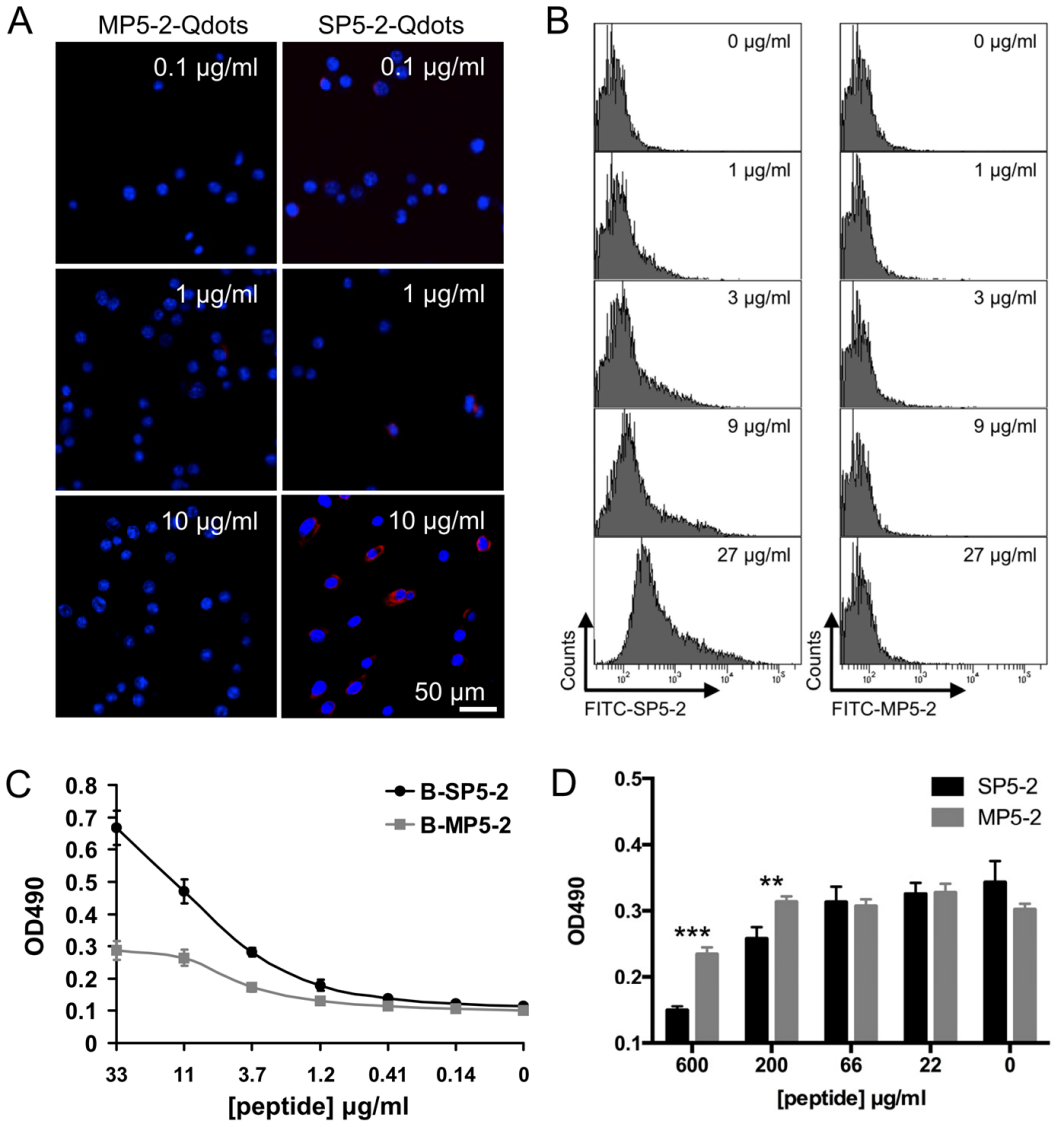
Verification of SP5-2 binding to a mouse (LL/2) and human (H460) lung cancer cell lines. (A) Binding of peptide-conjugated Qdots to tumor cells *in vitro* is peptide-sequence specific. The ability of quantum dots labeled with SP5-2 and MP5-2 to bind to the surface of LL/2 cells was examined (Scale bar, 50 µm). Nuclear staining was performed using DAPI. (B) LL/2 cells were incubated with SP5-2 FITC-conjugated (FITC-SP5-2) or MP5-2 FITC-conjugated (FITC-MP5-2) peptides at increasing concentrations. The FITC-SP5-2 treated LL/2 cells showed increasing flurescence intensity at increasing peptide concentrations, but no such correlation was observed in FITC-MP5-2 treated cells. (C) H460 cells were seeded in 96-well ELISA plates. Biotinylated SP5-2 (B-SP5-2) and biotinylated MP5-2 (B-MP5-2) were used to test the peptide binding assay. (D) Peptide competitive inhibition assay with the unlabeled SP5-2 peptide. The black bars showed the binding inhibition of the biotinylated peptide (B-SP5-2, 10 µg/ml) by the unlabeled SP5-2 peptide (from 22 to 600 µg/ml). The gray bars showed the binding inhibition of B-SP5-2 by the MP5-2 peptide. The statistic value was compared to control peptide. **, p value <0.01; ***, p value <0.005.

To further investigate the binding ability of SP5-2 to Lewis lung carcinoma (LL/2) cells, the cells were cultured and incubated with peptide-coated quantum dots (Qdots). SP5-2-Qdots were observed on the surface of LL/2 cells, whereas no signal was observed for cells incubated with MP5-2-Qdots (Figure 3A). This experiment showed that the SP5-2 is required for the binding of Qdots to cells *in vitro*. We also confirmed that SP5-2 significantly enhanced the binding affinity to LL/2 cells relative to the MP5-2 in a dose-dependent manner using FACS staining (Figure 3B). The examination of the SP5-2 binding assay with H460 showed that the binding curve ranged from 0.14 to 33 µg/ml and that the minimum binding activity started at 1 µg/ml (Figure 3C). Based on the predicted binding motif, we investigated how mutations within the peptide sequence affect its binding properties. The results showed that by changing WTY motif of SP5-2 [11] to GGG of MP5-2, mutant SP5-2 exhibited a remarkable loss of binding activity (Figures 3A and 3C). Biotin-labeled SP5-2 binding to H460 cells could be competitively inhibited by unlabeled SP5-2 but not by MP5-2 (Figure 3D). These results indicate that SP5-2 exhibits the specific affinity to bind to lung cancer cells.

### Enhanced endocytosis of SP5-2-LD increase therapeutic efficacy in a syngenic mouse model

**Figure 4:**
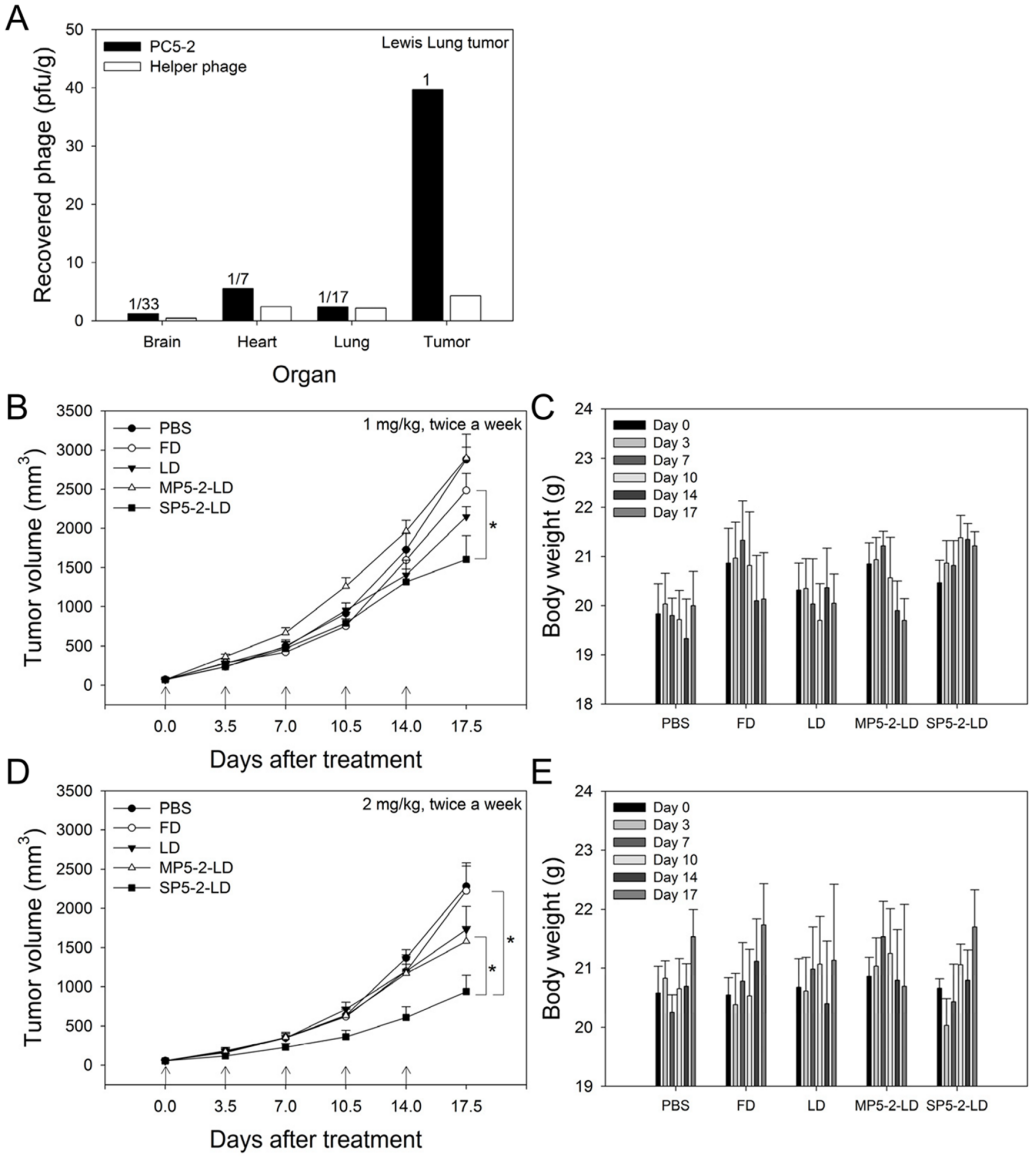
SP5-2-LD increases therapeutic efficacy in a syngenic animal model. (A) C57BL/6 mice bearing syngenic LL/2 xenografts were injected i.v. with PC5-2 (phage displaying SP5-2) or control phages, and phage titers were examined in tumor tissue and the indicated organs. (B) Eight-week-old C57BL/6 mice bearing LL/2 xenografts were injected i.v. with different doxorubicin formulations twice a week for 17 days. Mice were treated with either 1 mg/kg or 2 mg/kg of SP5-2-LD, MP5-2-LD, LD, FD or PBS. In the 1 mg/kg treatment of doxorubicin, tumor size (B) and body weight (C) were measured at the time point of injection and at the indicated times thereafter. (D, E) indicate the tumor size and body weight of 2 mg/kg treatment, respectively. All data shown are on the mean averages of six mice per group. (*, p value <0.05.)

In our previous study, SP5-2 has been proven beneficial for the therapeutic index of liposomal drugs in a xenograft animal model [11]. To further determine the treatment potential of targeting SP5-2-LD in a more accurate representation of the tumor's microenvironment, a syngenic model involving the transplantation of a tumor to an organism with the same genetic background was used. To develop a syngenic animal model, we first investigated the tumor targeting ability of SP5-2 to Lewis lung carcinoma (LL/2) cells. We confirmed the tumor targeting ability of SP5-2 to LL/2 cells by injecting PC5-2 phage (phage-displayed SP5-2) into LL/2 tumor-bearing mice (Figure 4A). PC5-2 showed homing ability to tumor masses, where it was present in concentrations 7-fold higher than that in the control organs. Control helper phages did not show any specific targeting to tumor tissues (Figure 4A).

To explore whether SP5-2 could be used to improve the efficacy of chemotherapeutics in a syngenic animal model, C57BL/6 mice bearing LL/2-derived tumors were treated with SP5-2-LD, MP5-2-LD, LD, Free Doxorubicin (FD), or equivalent volumes of phosphate-buffered saline (PBS). All the formulations were injected i.v. at a doxorubicin dosage of either 5 or 10 mg/kg (twice a week at 1 or 2 mg/kg for a total of five injections). The tumors in mice that received SP5-2-LD were noticeably smaller than those in the MP5-2-LD, LD, FD, and PBS groups at the cessation of treatment (Figures 4B and 4D). The tumors in mice treated with 1 and 2 mg/kg of FD were 1.55- and 2.38-fold larger than those in the equivalent SP5-2-LD-treated mice, respectively. No significant loss in body weight was observed following treatment with either concentrations of SP5-2-LD (Figures 4C and 4E). These data reveal that SP5-2-LD increases therapeutic efficacy as compared with FD or LD in our C57BL/6 syngenic animal model.

### Therapeutic efficacy of SP5-2-mediated targeting liposomes in metastatic tumors

**Figure 5:**
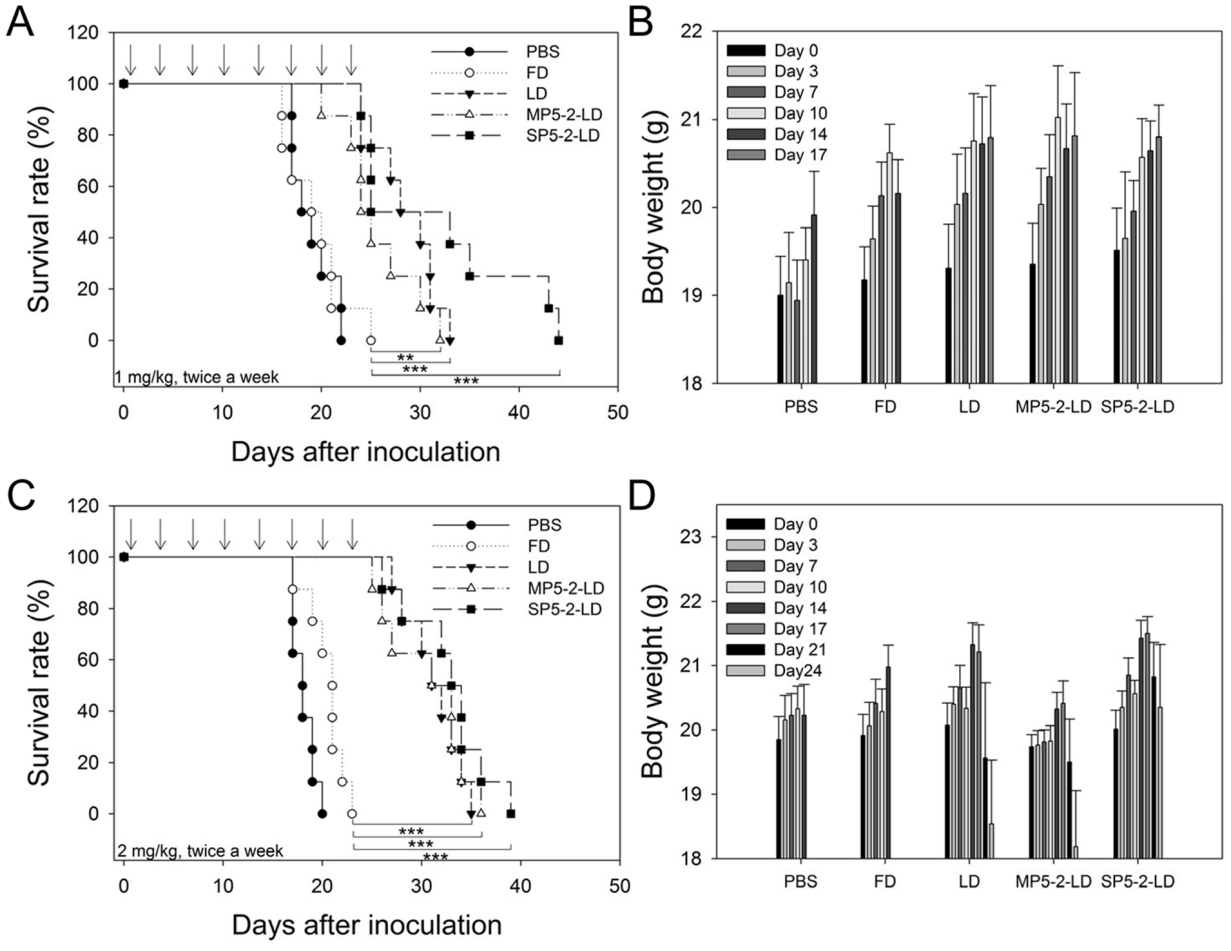
SP5-2-LD enhances survival in a metastatic tumor animal model. C57BL/6 mice were injected i.v. with 1×10^5^ LL/2 cells, incubated for 24 hours, and then treated with different doxorubicin formulations via i.v. injections twice a week. Mice were treated with either 1 mg/kg or 2 mg/kg of SP5-2-LD, MP5-2-LD, LD, FD or PBS (n = 8). The survival rate is shown in (A, C) for 1 and 2 mg/kg treatment, respectively. Body weight is shown in (B, D) for 1 and 2 mg/kg treatment, respectively. (**, p value <0.01; ***, p value <0.005.)

Aggressive, rapidly growing tumors often invade surrounding tissue and metastasize, and are always associated with poor prognosis. In the development of metastases, tumor cells enter into the circulation, followed by adhesion, extravasation and the establishment of metastatic lesions [42]. We further investigated whether the SP5-2-LD could increase the median overall survival of metastatic tumor-bearing mice by generating a Lewis lung carcinoma (LL/2) metastatic animal model. LL/2 cells were injected i.v. into C57BL/6 mice. Twenty-four hours after injection, C57BL/6 mice bearing circulating LL/2 cells were treated with SP5-2-LD, MP5-2-LD, LD, FD, or equivalent volumes of PBS, through i.v. injection at a total doxorubicin dosage of 8 mg/kg (twice a week at 1 mg/kg for a total of eight injections). The median overall survival of tumor-bearing mice treated with SP5-2-LD, MP5-2-LD, and LD were significantly higher than those of FD- and PBS-treated groups (Figure 5A). A similar result was observed when the doxorubicin dose was doubled (16 mg/kg; injected twice a week at 2 mg/kg for a total of eight injections) (Figure 5C). The median overall survival of tumor-bearing mice after treatment with SP5-2-LD, MP5-2-LD, LD, FD, and PBS groups were 33.5, 32, 31.5, 21, and 18 days, respectively (Figure 5C). However, tumor-bearing mice treated with a high-dose of MP5-2-LD or LD were observed to decrease in body weight as compared to the SP5-2-LD group, suggesting a toxic side-effect of the formulation (Figure 5D). These results demonstrate that SP5-2 slightly increases the therapeutic efficacy of liposome-encapsulated doxorubicin while reducing its toxicity.

### Therapeutic efficacy of SP5-2-LD in an orthotopic animal model

**Figure 6:**
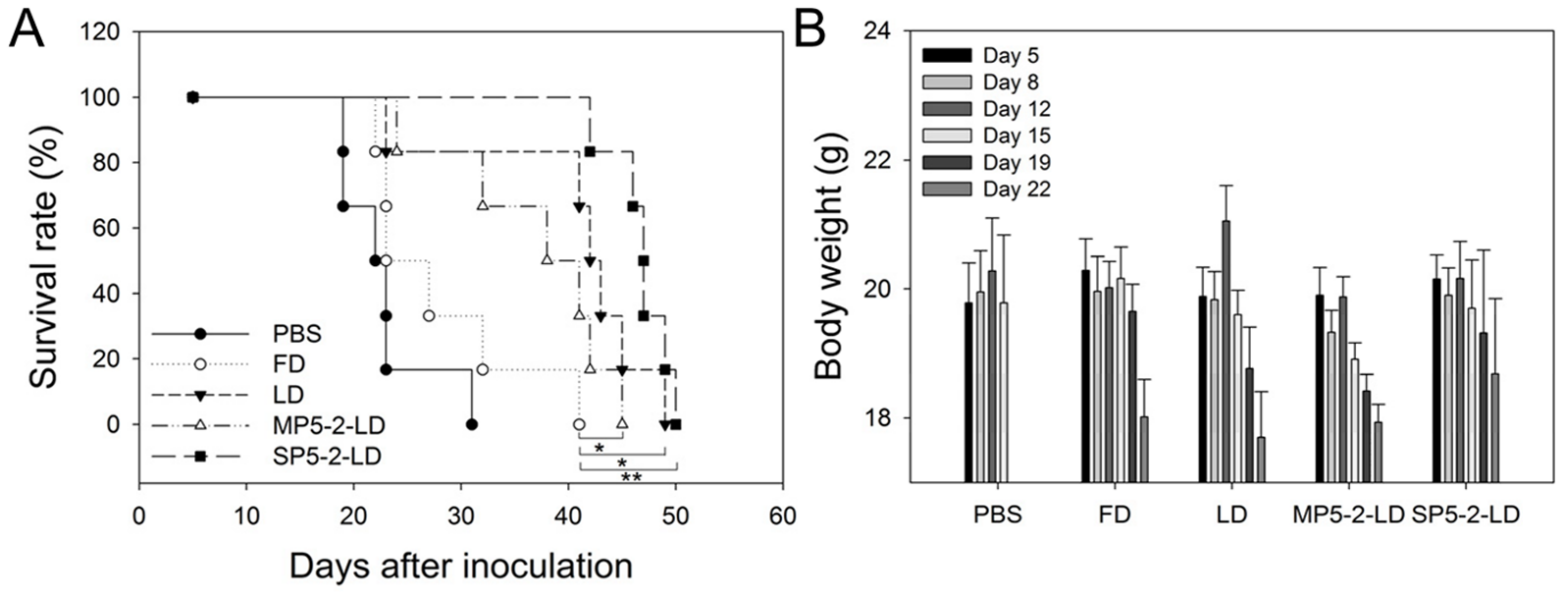
SP5-2-LD enhances survival and reduces side-effects in an orthotopic tumor model. The lungs of SCID mice were injected with 5×10^5^ H460 cells. Mice were treated with SP5-2-LD, MP5-2-LD, LD, FD or PBS (n = 6, 1 mg/kg, twice a week) five days after injection. Median overall survival is shown in (A). Body weight is shown in (B). (*, p value <0.05; **, p value <0.01).

Our primary goal of modern cancer therapy is to increase the therapeutic index of liposomal drugs by enhancing efficacy while reducing toxicity. When tumor-bearing mice were treated with SP5-2-LD, the results showed that the mice had the smallest tumor sizes and more stable body weights, indicating that SP5-2 may increase therapeutic index by enhancing efficacy and reducing toxicity (Figures 4 and 5). To further confirm that SP5-2-mediated liposomes increase the therapeutic index, we established an orthotopic mouse model of lung cancer by implanting H460 cells into lung tissues. SP5-2-LD, MP5-2-LD, LD, FD, or equivalent volumes of PBS were injected i.v. (total doxorubicin dosage of 8 mg/kg; twice a week at 1 mg/kg for eight injections) five days after orthotopic injection of lung cancer cells. The mean life spans of the SP5-2-LD, MP5-2-LD, LD, FD, and PBS groups were 47, 39.5, 42.5, 25, and 22.5 days (Figure 6A), respectively. Compared to the other groups, mice treated with SP-5-2-LD not only lived longer, but also underwent the smallest change in body weight (Figure 6B).

### Biodistribution of SP5-2-conjugated targeting liposomes

**Figure 7:**
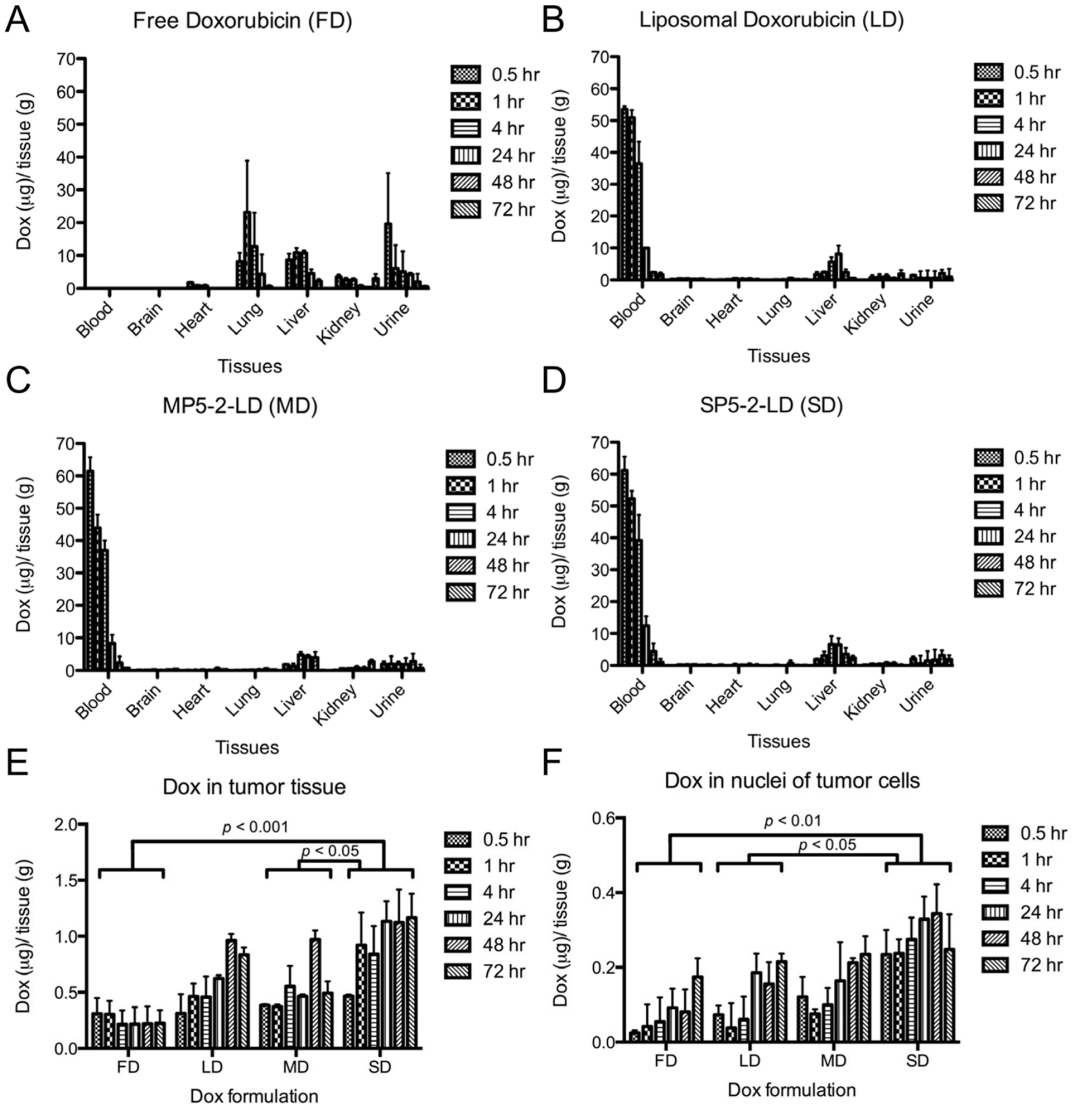
Biodistribution of doxorubicin in mice treated with different anti-cancer drug formulations. Doxorubicin formulations were injected into the tail vein of H460 tumor-bearing mice. Various organs were collected at the indicated time points, and the doxorubicin concentration was determined (bars, S.D.). The concentrations in each organ are normalized to that in tumor at the equivalent time point, and are presented as the average of three experiments. The biodistribution of free doxorubicin (A), liposomal doxorubicin (LD) (B), MP5-2-LD (C), and SP5-2-LD (D) in each organ of the treated mice are shown. (E) Comparison of doxorubicin accumulation in tumor tissue. Tumor tissues were collected, homogenized, and quantified for fluorescence. (F) Cellular nuclei were isolated from the tumor tissues, and the concentration of nuclear doxorubicin was determined by measuring the intrinsic auto-fluorescence signal of doxorubicin. All data are the mean averages of data from three mice per group.

Accumulating data have revealed that the endocytotic ability of SP5-2 results in effective delivery and minor toxicity. To comprehensively understand the pharmacokinetics of SP5-2-LD *in vivo*, we initially asked whether conjugation of the NSCLC-targeting peptide SP5-2 to liposomal doxorubicin (LD) would affect the latter's biodistribution. To this end, the biodistribution of LD, SP5-2-LD, MP5-2-LD, and FD were estimated by measuring the intrinsic auto-fluorescence signal of doxorubicin in mice with H460 lung cancer xenografts. Because doxorubicin (FD) is a small-molecule chemotherapeutic agent, it will distribute throughout the whole body indiscriminately via circulation, leading to poor pharmacokinetic profile with quick elimination pattern and inefficient targeting of tumors (Figures 7A and 7E). Incorporating doxorubicin into liposomes (LD), however, greatly improved its pharmacokinetic profile as compared to FD (Figure 7 and Table 1). LD and MP5-2-LD increased drug accumulation in tumor (Figure 7E), but decreased drug penetration in organs (such as heart, lungs, liver, and kidneys) (Figures 7B and 7C). SP5-2-LD resulted in more effective penetration of doxorubicin, and greater accumulation of the drug in tumor tissues (Figures 7D and 7E).

**Table 1:** Tumor pharmacokinetics of free doxorubicin versus liposomal doxorubicin formulations.

Formulation (2 mg/kg)	Tumor*	Nuclei	Heart	Lung	Liver	Kidney	Blood
Free doxorubicin	8.5	5.5	26.3	421.2	293.6	87.2	5.9
Liposomal doxorubicin	52.8	12.4	20.7	17.5	308.5	67.5	789.4
MP5-2-LD	46.4	12.8	19.2	19.7	243.5	68.6	734.2
SP5-2-LD	94.8	26.2	12.6	12.4	254.6	75.6	939.4

Note. MP5-2-LD  =  mutant SP5-2 conjugated liposomal doxorubicin; SP5-2-LD  =  SP5-2 conjugated liposomal doxorubicin. AUC values were calculated using the doxorubicin dosages in each organ from different time points, including 0, 0.08, 0.25, 0.5, 1, 4, 24, 48, and 72 hours. This experiment was performed in three individual mice.

*AUC_0-72 h_ (µg·h/g)

To understand the SP5-2-enhanced endocytosis of liposomal doxorubicin *in vivo* and to assess the bioavailability of the liposomal drugs, we used nuclear doxorubicin accumulation as an indicator of drug-mediated cytotoxicity. As shown in Figure 7F, the concentration of doxorubicin in SP5-2-LD-treated group was overall higher than those in LD- and MP5-2-LD-treated groups. Moreover, the area under the concentration-time curve (AUC_0–72 hours_) of doxorubicin in tumor tissues was 11.2-, 1.8- and 2.0-fold higher in the SP5-2-LD group as compared to the FD, LD and MP5-2-LD groups, respectively (Table 1). The AUC_0–72 hours_ of bioavailable doxorubicin (i.e., bound to nuclei) was 4.8-, 2.1- and 2.0-fold higher in the SP5-2-LD group than that in the FD, LD and MP5-2-LD groups, respectively (Table 1). Finally, the AUC_0-72 hours_ of doxorubicin circulating in the blood was 159.2-, 133.8- and 124.4-fold higher in the SP5-2-LD, LD and MP5-2-LD groups than that in the FD group, respectively (Table 1).

## Discussion

Many common cytotoxic drugs used for chemotherapy have several disadvantages, including poor pharmacokinetic profiles, narrow therapeutic windows and indiscriminate entry into both healthy and malignant tissues. The effectiveness of chemotherapy is severely limited by the toxicity of the anticancer agents to normal cells. In addition, some chemotherapeutic agents exhibit organ-specific toxicities, resulting in severe side effects [43], [44], [45]. Several approaches have been developed to enhance the selectivity of anticancer drugs towards tumors. In recent studies, doxorubicin treatment has been shown to be associated with severe cardiotoxicity and hepatotoxicity; however, these side effects could be reduced with PEGylated liposomes, which increased the tumor localization of the nanoparticle formulation (Table 1) [17]. Although PEGylated liposomes have several advantages, their usefulness is limited by their reliance on passive targeting. A promising new strategy is to encapsulate anticancer drugs in liposomes conjugated with moieties, such as antibodies or peptides, which can target particular types of tumor cell or tumor vasculature [12], [22]. Liposomal drugs that are not internalized by cancer cells have been found to accumulate substantially in tumor tissues, without exhibiting an antitumor effect [46], [47]. Because anti-cancer drugs cannot diffuse through the intact liposomal membrane, they tend to stay inside the PEGylated liposome and thus display little therapeutic activity. To overcome this problem, we identified lung cancer cell-targeting peptides with two important characteristics: highly specific binding to lung cancer cells, and the ability to stimulate endocytosis for successful delivery of liposomal drugs into cells. In this study, we found that the encapsulated drugs are released through receptor-mediated endocytosis into the cytoplasm of the cancer cells (Figures 1 and 2). Receptor-mediated endocytosis, an essential step for many targeted therapeutics, can potentially increase the bioavailability of antitumor drugs [48]. SP5-2 is demonstrated to enable internalization *in vitro* (Figure 1). Endocytosis delivers targeting liposomes to the endosomes (Figure 2A), where the pH is 5.9–6.0 [49]. The molecules are subsequently transferred to lysosomes (Figure 2A), where the pH is even lower, at 5.0 [50]. The lysosomes also contain enzymes that break down the cargo. As such, internalized liposomes are broken down to release encapsulated drugs into the intracellular space.

The rate of drug release from the nanoparticles can be controlled to match the pharmacodynamic profile of the drug. This can be achieved through three approaches: (a) conjugating a targeting ligand to increase the rate of intracellular delivery and the bioavailability of the drug; (b) incorporating a pH sensitive component in the formulation to facilitate drug release from the endosome; or (c) triggering drug release locally through a physical method at the target tissue [51]. In this study, we aimed to develop ligand-mediated targeting liposomes to improve the tumor site-specific action of the delivery system. In our three different animal models, both the non-targeting and the targeting liposomes provided a biodistribution and pharmacokinetic advantage relative to free drug solution in therapeutic efficacy. Liposomes conjugated to ligands that target internalizing antigens have been found to reach tumor cells with higher specificity, increase the bioavailability of the anticancer agent in solid tumors, and have greater cytotoxicity (Figures 4–6). This illustrates that these liposome formulations were identical in the quantitative and qualitative biodistribution profiles due to the beneficial delivery of passive targeting. Although the SP5-2-LD did not significantly enhance the therapeutic efficacy and prolong the life span, they increased the bioavailable doxorubicin accumulation in tumor cell nuclei by 4.8-fold and 2.1-fold compared to FD and LD, respectively (Figure 7 and Table 1). This phenomenon is very important to consider when designing and evaluating drug delivery systems. The minute difference between these two formulations, LD and SP5-2-LD (surface modification with 12-mer SP5-2 peptide), is enough to alter the biodistribution and the pharmacokinetic profile of the PEGylated liposomes. These results are significant because they prove the concept that the active targeting ligands may confer a clinical advantage for the delivery of nanocarriers. In our previous study using different cancer cell lines, we found that targeting liposomal doxorubicin was able to significantly increase the therapeutic efficacy and the life span of tumor bearing mice [11], [22]. In this study, the tumor-bearing mice developed drug resistance against doxorubicin, leading to the decreased benefits observed using this peptide-mediated drug delivery system. Hence, we believe that the therapeutic efficacy as shown in the animal models is highly dependent on the sensitivity of the cancer cells to doxorubicin and the type of animal models used. In the future, delivery of anticancer drugs with multiple mechanisms for killing cancer cells (i.e., doxorubicin + other chemotherapeutic agents) might markedly increase the therapeutic efficacy by prolonging the overall survival.

An important and often overlooked aspect of drug delivery is the clinical translation. Systemic administration of cytotoxic drugs is the primary treatment strategy for cancer patients; however, it typically induces lethal toxicity to normal tissues in a wide range of treatment regimens [52], [53]. In clinical therapies, the dose of cytotoxic agents are limited by patient tolerance and are usually at or near the maximum tolerated dose (MTD) to reach the largest possible killing activity to tumor cells. Although most research efforts in cancer therapies are focused on combinations of agents, doses, and dose schedules that maximally kill tumor cells, the serious side effects still cause the toxicity to the host. Thus, the fundamental goal is to induce lethal toxicity in the largest possible number of tumor cells while minimize the toxicity accompanied by the treatment. In this study, the antitumor efficacy of the targeting liposomes, which correlated with the level of tumor accumulation, was superior to that of the free drug as well as to that of drug encapsulated in non-targeting liposomes. We observed several important attributes of SP5-2-LD, including extended pharmacokinetic data, increased drug bioavailability in cancer cells, improved therapeutic efficacy, and reduced side effect. Therefore, combining drug delivery systems with ligands that specifically bind to cancer cells (especially cancer stem cells) and tumor blood vessels presents a preferred cancer treatment strategy due to its potential for enhancing the therapeutic index. Furthermore, internalization of liposomes through receptor-mediated endocytosis enables the release of drugs into the intracellular space of cancer cells, which may overcome efflux pump-mediated drug resistance. The benefit of peptide-mediated liposomes can be expected to be more widely adopted in the clinical treatment of cancer in the future.

The liposome-conjugated peptides, which are both well-exposed on the surface and in appropriate conformation for binding, are promising tools for selective delivery of nanoparticles to the cellular targets [54]. In addition, the number of conjugated peptide will also influence the effect of targeting liposomes. The uptake of liposomes might increase for liposomes with higher number of peptides. However, it is dependent on different cancer cells because of the different receptor density on the cell surface and the binding affinity of peptide [55], [56]. Also, the pharmacokinetics and pharmacodynamics might be changed by different number of conjugated peptides. In this study, 500 peptides conjugation is used for the entire experiment. Although the animal studies showed statistical significance of SP5-2-mediated liposomes compared to non-targeting liposomal drugs in both drug accumulation and therapeutic efficacy, the ideal number of peptide required to achieve optimal therapeutic index will need to be further evaluated.

The development of novel drug delivery systems that improve pharmacokinetics and bioavailability of the drugs, while reducing their side effects, is urgently needed. In this study, we found that coupling SP5-2 to liposomes increased the accumulation of cytotoxic drugs in both tumor tissues and within cancer cells, resulting in enhanced therapeutic efficacy in three different animal models. The conjugation of internalizing ligands to PEGylated liposomes allows the encapsulated contents to be delivered to the cytosol via receptor-mediated endocytosis, thereby increasing the therapeutic index by enhanced regulation of drug release. Targeted delivery through highly specific peptide bears tremendous promise in improving both the efficacy and the safety profiles of liposomal drug delivery systems. Areas for future clinical development include the optimization of targeting ligands, conjugation methodologies, liposome design, and CMC (Chemistry, Manufacturing and Control) management.
